# Feasible Nipple Preservation Techniques for Breast Cancer Under the Nipple-Areolar Complex: A Case Report

**DOI:** 10.7759/cureus.85886

**Published:** 2025-06-12

**Authors:** Koki Yamamoto, Shoji Oura

**Affiliations:** 1 Department of Surgery, Kishiwada Tokushukai Hospital, Kishiwada, JPN

**Keywords:** breast cancer under the nipple, nipple base resection, nipple preservation, thick skin flap, venous return from the nipple

## Abstract

We present our nipple preservation techniques for breast cancer under or near the nipple-areolar complex through actual surgical procedures in a 59-year-old patient with node-negative luminal breast cancer. Our main surgical points for nipple preservation against juxta-nipple breast cancer were as follows. First, we made an extended peri-areolar incision. Second, we left the subcutaneous fat near the nipple in a thick flap manner on skin flap formation to ensure venous drainage from the nipple-areolar complex. Then, we incised the distal mammary gland with safety margins, bluntly dissected the retromammary space to manually palpate the breast cancer in order to avoid off-center resection, forwarded the mammary gland incision cranially and caudally toward the nipple, and completely skeletonized the mammary gland just under the nipple. Finally, we resected the sub-nipple mammary gland at the lactiferous level with the nipple slightly depressed below the surrounding skin by pulling the breast tissue to be resected, leading to maximal removal of the mammary gland, including some intra-nipple mammary ducts. After the pathological confirmation of negative surgical margins at the nipple base and node negativity, the patient received postoperative radiation therapy to the conserved breast and has been well for seven months on letrozole therapy. Breast surgeons should be familiar with these surgical procedures for feasible nipple preservation in juxta-nipple breast cancer.

## Introduction

The advent of breast-conserving therapy has greatly benefited many breast cancer patients [1,2]. Breast-conserving therapy is a treatment that does not aim to simply preserve some part of the breast, but to achieve favorable cosmetic outcomes after operation. Postoperative cosmetic outcomes of the breast can be affected by various factors, such as tumor locations and breast sizes. Postoperative breast size and nipple symmetry, especially, have a significant impact on cosmetic outcomes. Therefore, preservation of the nipple-areolar complex (i.e., the nipple and areola) plays a very important role in excellent post-operative cosmetic outcomes of the breast.

Breast cancer located just under the nipple-areolar complex generally suggests a short distance between the nipple and the tumor. Furthermore, many breast surgeons perform breast-conserving surgery in a manner to vertically resect the mammary gland and fat tissue around breast cancers [3]. Some breast surgeons, therefore, consider breast cancers located under or close to the nipple-areolar complex to be ineligible for breast-conserving therapy despite the absence of direct cancer cell extension to the nipple-areolar complex.

Nipple-areolar complexes have cancer foci, if present, not with invasive cancer, but with non-invasive cancer in the vast majority of breast cancer patients with nipple involvement. Therefore, if breast surgeons can remove as much of the ductal spread toward the nipple as possible, many breast cancer patients can benefit from nipple preservation. Unfortunately, however, there are currently no established surgical methods for appropriately resecting ductal spread toward the nipple.

We herein present our nipple preservation techniques for juxta-nipple breast cancer through actual surgical procedures in a patient with breast cancer located very close to the nipple-areolar complex.

## Case presentation

**Figure 1:**
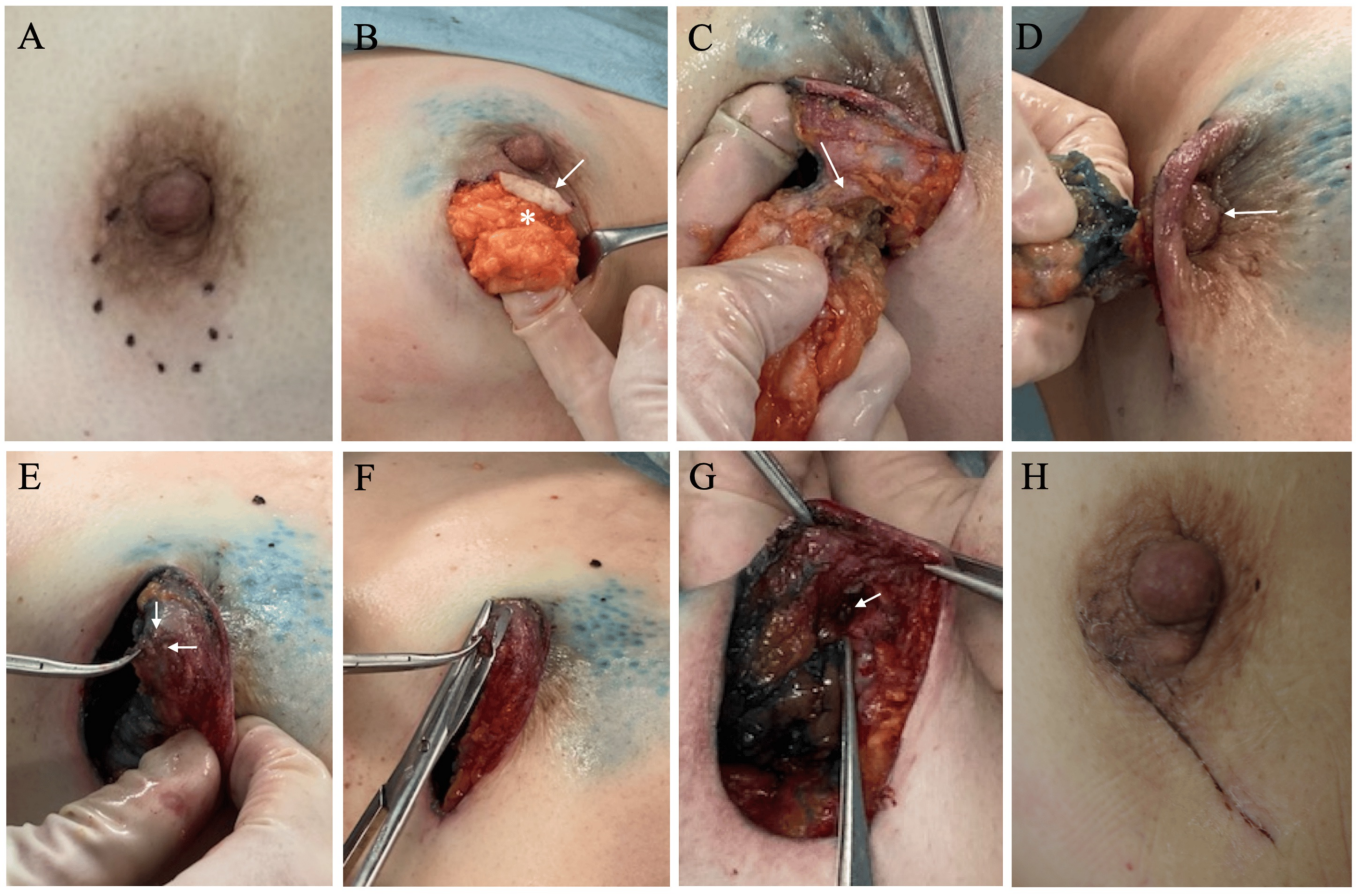
Operative procedures and nipple-areolar status seen from the lateral position (A) The tumor (dotted circle) located at least partially under the nipple-areolar complex. (B) After making a thin spindle skin incision (arrow) just above the tumor (asterisk) and incising the distal breast tissue with safety margins, we bluntly dissected the retromammary space to avoid off-center partial mastectomy. (C) After reaching the subnipple area, we circumferentially incised the subcutaneous fat tissue to skeletonize the subnipple mammary gland (arrow). (D) We cut the subnipple mammary gland in a condition that the nipple (arrow) was slightly depressed below the surrounding skin by pulling the mammary tissue scheduled to be resected. (E) After subnipple mammary gland resection, the inverted nipple had scarce intranipple mammary gland and several visible intranipple mammary duct stumps (arrows). (F) We resected the remaining small mammary gland tissue left in the nipple using scissors and submitted it for frozen section, showing no positive margins in the nipple. (G) After the completion of nipple base resection, we could find a small cavity beneath the nipple (arrow). (H) Two weeks after the operation, we could find favorable wound healing and an intact nipple-areolar complex.

A 59-year-old postmenopausal woman noticed a right painless breast mass near the nipple-areolar complex seven days before (Figure 1A) and was referred to our hospital for detailed examination.

**Figure 2:**
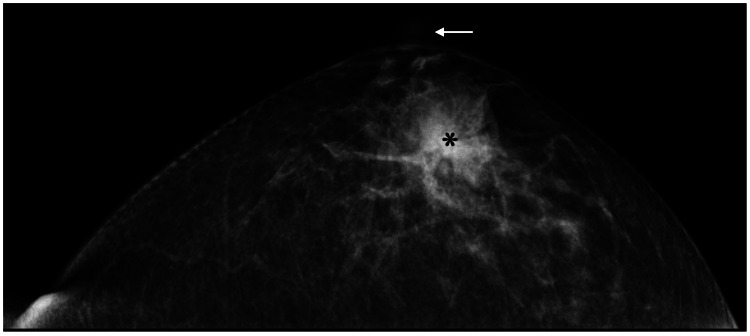
Mammography findings Mammography showed focal asymmetric density (asterisk) close to the nipple (arrow).

The patient's family history included breast cancer in her sister. Mammography only showed focal asymmetric density near the nipple (Figure 2).

**Figure 3:**
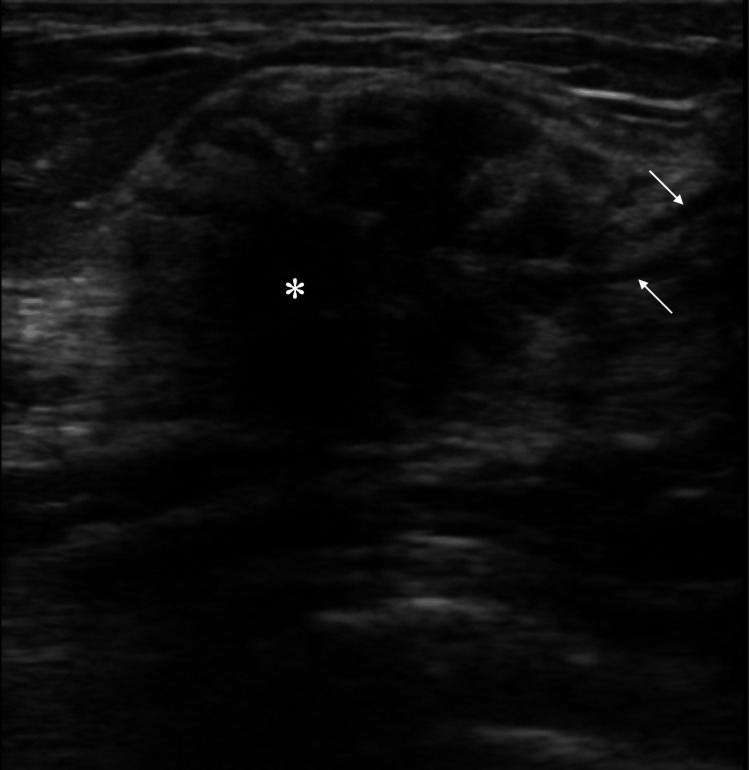
Ultrasound findings Ultrasound showed an oval mass (asterisk) and tubular hypo-echoic lesions (arrows), suggesting intraductal spread toward the nipple.

Ultrasound showed that an irregular mass had indistinct margins, a high depth-width ratio, low internal echoes, tubular low echoes just beside the mass, and heterogeneous posterior echoes that were attenuated or enhanced depending on the location (Figure 3).

**Figure 4:**
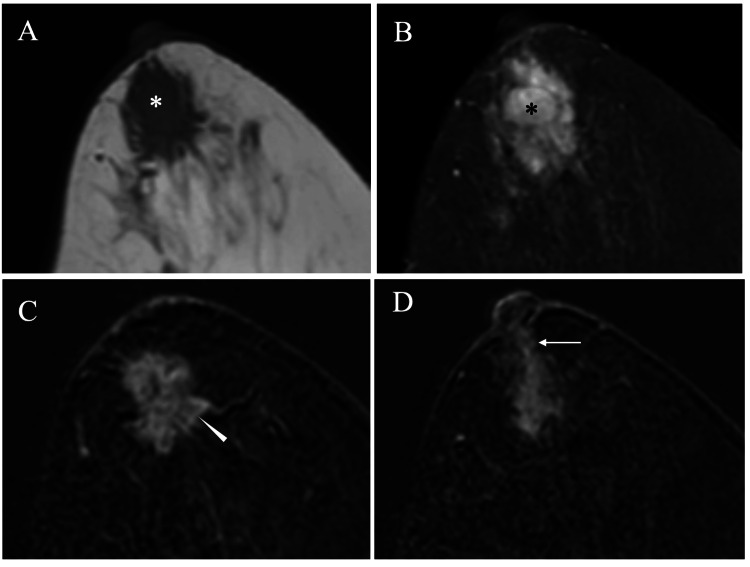
Magnetic resonance image (MRI) findings (A) MRI of the tumor showed low signals (asterisk) on T1-weighted images. (B) MRI of the tumor showed high signals (asterisk) on fat-suppressed T2-weighted images. (C) Subtraction MRI showed an oval mass with fast enhancement (arrowhead). (D) Subtraction MRI showed the presumed ductal spread (arrow) toward the nipple.

Magnetic resonance imaging (MRI) clarified that the tumor had fast enhancement in the initial phase, presumed ductal spread toward the nipple, and stromal invasion neither to the overlying skin nor to the nipple (Figure 4).

**Figure 5:**
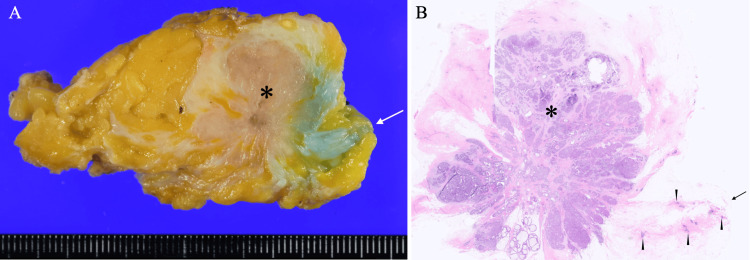
Pathological findings Bisected specimen showed a lobulated mass (asterisk) and subnipple mammary gland (arrow). Low magnified view showed sparse and scant cancer cell clusters (arrowheads) spreading toward the nipple base stump (arrow) in addition to the lobulated mass (asterisk).

A core needle biopsy pathologically showed atypical cells growing in tubular and trabecular fashions with interstitial connective tissue proliferation, leading to the diagnosis of invasive ductal carcinoma. Immunostaining showed that the tumor had positivity both for estrogen and progesterone receptors, negativity for human epidermal growth factor receptor type 2, and a Ki-67 labelling index of 15%. Due to the patient’s strong preference for nipple preservation, we tried to conserve the nipple-areolar complex in a manner to maximally resect the ductal spread toward the nipple in breast-conserving surgery after confirming the negative sentinel node on frozen section. Procedures of nipple preservation in breast-conserving surgery for juxta-nipple breast cancer were as follows. First, we made a skin incision in a manner to laterally extend the peri-areolar incision. Second, in a thick flap manner, especially around the nipple-areolar complex, we made skin flaps widely to facilitate the resection of the target tissue. Then, we started the partial mastectomy by incising the distal mammary gland with safety margins and thereafter bluntly dissected the retro-mammary space (Figure 1B). Next, we forwarded the full thickness incision of the mammary gland cranially and caudally toward the nipple base while avoiding off-center resection by palpating the mass, making complete skeletonization of the sub-nipple mammary gland at the lactiferous sinus level (Figure 1C). Finally, we performed nipple base resection with the nipple in a condition slightly depressed below the peri-areolar skin by grasping and pulling the mammary tissue to be resected (Figure 1D). These procedures allowed us to confirm the faint residual mammary tissue in the nipple (Figure 1E) and to submit the small intra-nipple tissue for an intra-operative surgical margin check (Figure 1F). After confirming no positive margins in the nipple on frozen section, the edges of the conserved breast were sutured to close the resection cavity, leading to the completion of partial mastectomy (Figure 1G). Post-operative pathological study showed that the tumor had atypical cells growing in cribriform and tubular fashions, sparse ductal spread of cancer cells toward the nipple, and negative but close margins at the nipple base on permanent sections (Figure 5).

The patient was discharged on the third day after operation without any events in the nipple-areolar complex (Figure 1H) and has been well for seven months on letrozole therapy after receiving radiotherapy to the conserved breast according to the St. Gallen International Consensus Guideline [4].

## Discussion

Small breast cancers near the nipple-areolar complex have four surgical options. The most common option is a mastectomy [5]. The next less common option is breast-conserving surgery with nipple-areolar complex resection [6]. Nipple-areolar reconstruction after the second surgical option is the third option [7]. The last option, actually done in this case, is to resect the ductal spread as much as possible, enabling the preservation of nipple-areolar complex [8].

Resection of the ductal spread near the nipple-areolar complex can cause nipple necrosis. Many breast surgeons, therefore, are reluctant to remove as much sub-nipple mammary gland tissue as possible because they mistakenly believe that nipple necrosis is caused by nipple ischemia. We, however, have already found that nipple necrosis occurs not by nipple ischemia but by nipple congestion [9]. In other words, extensive resection of the ductal spread, even including the intra-nipple ductal spread, does not cause nipple necrosis as long as nipple congestion is avoided. Intact venous return from the nipple warrants the avoidance of nipple congestion followed by nipple necrosis. Uninjured subcutaneous veins around the nipple-areolar complex ensure venous return from the nipple, which requires the preservation of subcutaneous fat around the nipple [9,10]. In fact, relatively thick subcutaneous fat was preserved after completion of the partial mastectomy in this case.

Additively resected very small tissue in the nipple was submitted for frozen section and postoperatively for permanent section. These pathological examinations naturally could not completely negate the presence of very small non-contiguous and non-invasive ductal carcinoma foci that might still remain in the preserved nipple. However, minute cancer residuals, even if present in the preserved nipple, seemed to be easily controlled by post-operative radiotherapy to the breast [11].

For safe and maximum resection of ductal spread, it is very important to resect the nipple base with the nipple in a fashion slightly depressed below the peri-areolar skin [9,10]. Partial inversion of the nipple can be achieved by pulling the breast tissue to be resected after skeletonizing the sub-nipple glandular tissue. These procedures can remove at least some of the intra-nipple glandular tissue without damaging the nipple envelope. Although breast cancer is located not just under the nipple-areolar complex but very close to the nipple-areola complex in this case, these surgical techniques can be applied to all breast cancer cases just under the nipple with stromal invasion, neither to the nipple nor to the overlying skin. Conversely, this operation cannot be performed for breast cancer that has invasive components up to the skin just above the tumor or to the nipple. These surgical procedures will enable more breast cancer patients, including even those with breast cancer just under the nipple, to safely benefit from nipple preservation in breast-conserving therapy.

## Conclusions

Marked improvements in MRI images have preoperatively enabled breast specialists to precisely detect ductal spread and therefore to less often apply nipple preservation even to breast cancer located away from the nipple. Our nipple-preserving techniques, however, should enable many breast surgeons to safely apply nipple preservation to patients with breast cancer very close to the nipple-areolar complex.

## References

[1] Veronesi U, Cascinelli N, Mariani L (2002). Twenty-year follow-up of a randomized study comparing breast-conserving surgery with radical mastectomy for early breast cancer. N Engl J Med.

[2] Fisher B, Anderson S, Bryant J (2002). Twenty-year follow-up of a randomized trial comparing total mastectomy, lumpectomy, and lumpectomy plus irradiation for the treatment of invasive breast cancer. N Engl J Med.

[3] Kasumi F, Iwase T, Yoshimoto M, Akiyama F, Sakamoto G (1995). Experience of quadrantectomy with axillary dissection without radiotherapy sustained by serial pathological examination for stage I breast cancer. J Cancer Res Clin Oncol.

[4] Curigliano G, Burstein HJ, Gnant M (2023). Understanding breast cancer complexity to improve patient outcomes: the St Gallen international consensus conference for the primary therapy of individuals with early breast cancer 2023. Ann Oncol.

[5] Maddox WA, Carpenter JT Jr, Laws HL, Soong SJ, Cloud G, Urist MM, Balch CM (1983). A randomized prospective trial of radical (Halsted) mastectomy versus modified radical mastectomy in 311 breast cancer patients. Ann Surg.

[6] Tausch C, Hintringer T, Kugler F, Schmidhammer C, Bauer M, Aufschnaiter M (2005). Breast-conserving surgery with resection of the nipple-areola complex for subareolar breast carcinoma. Br J Surg.

[7] Solis Bermudez U, Monroy Velázaquez MG, Lara Sampayo KL (2025). Modified C-V flap for nipple reconstruction. Cureus.

[8] Morita T, Oura S, Makimoto S (2022). Nipple preservation through partial nipple incision and partial areola resection for a breast cancer adjacent to the nipple-areolar complex. Case Rep Oncol.

[9] Yasuda K, Oura S, Makimoto S (2022). Successful enucleation of intra-nipple recurrence of breast cancer. Case Rep Oncol.

[10] Matsuki H, Oura S (2024). Feasible nipple preservation techniques for breast cancer with slight nipple retraction. Case Rep Oncol.

[11] Park CC, Mitsumori M, Nixon A (2000). Outcome at 8 years after breast-conserving surgery and radiation therapy for invasive breast cancer: influence of margin status and systemic therapy on local recurrence. J Clin Oncol.

